# Outcome Stratification in Non-ST-Segment Elevation Myocardial Infarction: Standardized Assessment of Left Ventricular Global Longitudinal Strain

**DOI:** 10.31083/RCM49528

**Published:** 2026-09-16

**Authors:** Yaoyuan Xiao, Xi Li, Qinghua Fu, Yi Tang, Mingxiang Tang

**Affiliations:** ^1^Department of Cardiology, Hunan Provincial People’s Hospital, The First Affiliated Hospital of Hunan Normal University, Clinical Medicine Research Center of Heart Failure of Hunan Province, Hunan Normal University, 410005 Changsha, Hunan, China; ^2^Clinical Research Department, Shenzhen Qianhai Shekou Free Trade Zone Hospital, 518067 Shenzhen, Guangdong, China

**Keywords:** myocardial infarction, non-ST-segment elevation myocardial infarction, echocardiography, left ventricular function, left ventricular global longitudinal strain, prognosis, adverse cardiovascular events

## Abstract

**Background:**

Left ventricular global longitudinal strain (LVGLS) is a sensitive index of left ventricular systolic function. However, the prognostic value of this metric in patients with acute non-ST-segment elevation myocardial infarction (NSTEMI) has not been fully established.

**Methods:**

We retrospectively enrolled patients with newly diagnosed NSTEMI at Hunan Provincial People’s Hospital between April 2020 and October 2023. All participants underwent transthoracic echocardiography (TTE) within 48 hours before coronary revascularization. Major adverse cardiovascular events (MACEs) were defined as readmission for unstable angina, non-fatal myocardial infarction, readmission for heart failure, or cardiac death.

**Results:**

We followed 181 patients with NSTEMI for a median of 599 days (interquartile range (IQR): 297–800). MACEs occurred in 40 patients. Patients who experienced MACEs had significantly worse LVGLS than those without events (–11.00 [–13.70, –7.77] vs. –16.10 [–18.90, –13.50]; *p* < 0.001). In multivariable Cox regression analysis, LVGLS independently predicted MACEs (hazard ratio (HR) = 1.310, 95% confidence interval (CI): 1.172–1.465; *p* < 0.001). For 12-month risk prediction, adding LVGLS to the baseline clinical model improved discrimination, increasing the area under the curve (AUC) from 0.668 to 0.835. Kaplan–Meier analysis showed a markedly higher risk of MACEs in patients with LVGLS >–13.75% than in those with LVGLS ≤–13.75% (HR = 7.463; 95% CI, 3.831–14.540; *p* < 0.001).

**Conclusions:**

LVGLS is a strong, independent predictor of adverse cardiovascular events and may improve early risk stratification in patients with NSTEMI.

## 1. Introduction

Non-ST-segment elevation myocardial infarction (NSTEMI) is a major cardiovascular condition. The proportion of NSTEMI among patients with acute myocardial infarction (AMI) increased from 33% in 1995 to more than 50% in 2015, accompanied by a rise in hospitalizations [1,2]. Despite standardized treatment strategies, many patients continue to experience major adverse cardiovascular events (MACEs), including unstable angina, non-fatal myocardial infarction, post-myocardial infarction heart failure, and death [3]. These outcomes impose a substantial public health burden; therefore, early risk stratification remains crucial in patients with NSTEMI.

Echocardiographic parameters of left ventricular (LV) systolic function are important for prognostic evaluation in patients with NSTEMI [4]. Left ventricular ejection fraction (LVEF) remains the most commonly used measure of LV systolic function. However, its assessment is often time-consuming and may show limited reproducibility in clinical practice [5,6]. Therefore, identifying a more efficient and reliable echocardiographic index for evaluating LV systolic function is clinically relevant.

Compared with LVEF, LV global longitudinal strain (LVGLS) is more sensitive for detecting subclinical LV systolic dysfunction because longitudinal myocardial fibers, located predominantly in the subendocardium, are more vulnerable to ischemia and early myocardial injury. Consequently, longitudinal strain may deteriorate before a measurable reduction in global pump function is reflected in LVEF [7,8].

LVGLS quantifies the relative change in myocardial length between end-diastole and end-systole using two-dimensional speckle-tracking echocardiography (STE). Numerous studies have shown that LVGLS can predict prognosis in patients with AMI [9,10]. However, the prognostic significance of LVGLS in patients with NSTEMI remains uncertain. Thus, this study aimed to investigate the value of LVGLS for risk stratification in patients with NSTEMI.

## 2. Methods

### 2.1 Study Population

This study enrolled patients with NSTEMI who were admitted to Hunan Provincial People’s Hospital between April 2020 and October 2023. The diagnosis of NSTEMI was based on established clinical guidelines [11]. Exclusion criteria included (1) inadequate imaging views, (2) concomitant non-cardiac disease with a life expectancy <1 year, and (3) moderate-to-severe valvular heart disease, defined according to guideline-based integrated echocardiographic assessment (*i*.*e*., at least moderate stenosis or regurgitation of any major valve) [12]. The study complied with the Declaration of Helsinki and was approved by the Ethics Committee of Hunan Provincial People’s Hospital (2024-143). Informed consent was obtained from all participants.

### 2.2 Echocardiography

LVGLS was assessed using the speckle-tracking strain analysis module integrated into the GE Vivid E90 ultrasound system (GE Healthcare, Chicago, IL, USA). All measurements were performed in accordance with current guidelines and consensus recommendations [13,14].

#### 2.2.1 Image Acquisition

Standard apical four-chamber, apical two-chamber, and apical long-axis views were obtained within 48 hours before coronary revascularization. Imaging parameters were optimized to clearly visualize the left ventricular endocardial and epicardial borders. The frame rate was set to at least 40 fps, and image depth was adjusted to include the entire left ventricle without excessive background interference. Three consecutive cardiac cycles were recorded, including for patients with arrhythmia.

#### 2.2.2 Region of Interest (ROI) Definition

After selecting the AFI functional module, the ROI was automatically set to encompass the myocardial wall between the endocardium and epicardium. Manual adjustments were performed to (a) exclude papillary muscles and pericardial tissue to prevent tracking interference; (b) adjust the ROI width to 5–8 mm to align with myocardial thickness; (c) ensure that the ROI extended from the mitral annulus to the left ventricular apex.

#### 2.2.3 Speckle Tracking and Quality Control

The software autonomously tracked myocardial acoustic speckles throughout the cardiac cycle. For segments with poor tracking, such as endocardial border deviation or speckle loss, tracking quality was visually reviewed, and corrections were made by repositioning the ROI or adjusting the tracking window in accordance with current strain echocardiography recommendations [12].

#### 2.2.4 LVGLS Calculation

Peak systolic (PS) longitudinal strain was assessed in all 17 LV segments: 6 from the apical four-chamber view, 6 from the apical two-chamber view, and 5 from the apical long-axis view. LVGLS was calculated as the average PS across these 17 segments.

### 2.3 Clinical Assessment and Follow-Up

Baseline data included demographic characteristics, biochemical parameters, Killip classification, and coronary angiography findings. The Modification of Diet in Renal Disease (MDRD) equation was used to estimate glomerular filtration rate (eGFR): eGFR = 175 × [Scr (mg/dL)]^–1.154^ × age^–0.203^ × 0.742 (if female) [15]. The Global Registry of Acute Coronary Events (GRACE) risk score was calculated from eight admission variables: age, heart rate, systolic blood pressure, Killip classification, creatinine level, cardiac arrest at presentation, troponin elevation, and ST-segment changes on ECG. All enrolled patients were followed up by telephone at 1, 3, 6, and 12 months, and every 6 months thereafter. A MACE was defined as readmission for unstable angina, non-fatal myocardial infarction, readmission for heart failure, or cardiac death. Follow-up ended on January 6, 2024. Only the first event was considered in the analysis.

### 2.4 Statistical Analysis

Continuous variables with a normal distribution are presented as the mean ± standard deviation (SD), whereas non-normally distributed variables are presented as the median and interquartile range (IQR). Group comparisons were performed using analysis of variance (ANOVA), Student’s *t*-test, or the Mann–Whitney U test, as appropriate. Categorical variables are presented as percentages (n (%)) and were compared using the chi-square test. Bivariate correlations were assessed using Pearson or Spearman correlation analysis, as appropriate. Receiver operating characteristic (ROC) curves were used to evaluate prognostic performance and identify optimal cutoff values. Survival analyses were performed using Kaplan–Meier curves and univariate and multivariable Cox proportional hazards models, with the multivariable Cox models fitted using the enter method. To assess potential multicollinearity between LVGLS and LVEF, a variance inflation factor (VIF) analysis was performed, followed by sensitivity analyses using Cox models including LVEF alone and both LVGLS and LVEF simultaneously. Incremental prognostic value in addition to the baseline clinical model, including GRACE score, diabetes, and Killip classification, was assessed using the concordance index, 12-month area under the curve (AUC), continuous net reclassification improvement (NRI), and integrated discrimination improvement (IDI). Internal validation of the LVGLS threshold was performed using bootstrap resampling. As a sensitivity analysis to minimize information loss from dichotomization, LVGLS was additionally modeled as a continuous variable, and an exploratory restricted cubic spline analysis was performed to assess the dose–response relationship between LVGLS and MACE risk. A two-sided *p* < 0.05 was considered statistically significant. All statistical analyses were performed using SPSS software (version 25.0; SPSS Inc., Chicago, IL, USA).

## 3. Results

### 3.1 Clinical Characteristics

Initially, 215 patients were screened for inclusion. We excluded 21 patients because of inadequate image quality, 3 patients due to severe mitral regurgitation, 1 patient with an advanced tumor and multiple organ failure with a life expectancy of <1 year, and 9 patients who were lost to follow-up. Consequently, 181 participants were included in the final analysis (Fig. 1). The median follow-up period was 599 days (range, 10.0–26.7 months). The cohort comprised 44 females (24.3%) and 137 males (75.7%), with a mean age of 62.51 ± 10.45 years (Table 1). Forty patients experienced MACEs, including heart failure, unstable angina, non-fatal reinfarction, and cardiac death.

**Fig. 1.**
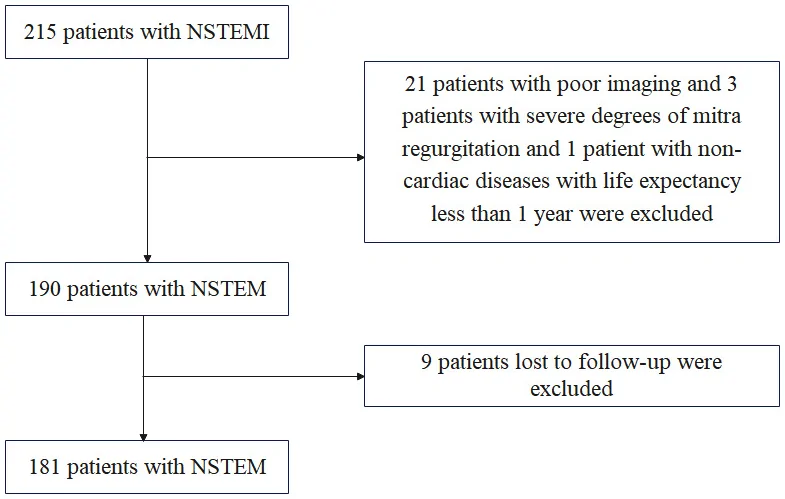
**Flowchart of patients in our study**. NSTEMI, non-ST-segment elevation myocardial infarction.

**Table 1.** **Clinical characteristics of the study cohort**.

Clinical characteristics			Total (n = 181)	Non-events (n = 141)	Events (n = 40)	*p*-value
Male, n (%)			137 (75.7)	106 (75.2)	31 (77.5)	0.762
Age, year			62.51 ± 10.45	62.60 ± 10.37	62.23 ± 10.85	0.844
BMI, kg/m^2^			24.03 [21.81, 26.14]	23.94 [21.94, 26.14]	24.14 [21.66, 26.08]	0.954
Heart rate, per/min			76.00 [69.00, 86.00]	76.00 [68.00, 85.00]	77.50 [71.00, 91.00]	0.206
Systolic blood pressure, mmHg			126.73 ± 19.06	125.72 ± 18.93	130.28 ± 19.35	0.183
Smoking, n (%)			103 (56.9)	78 (55.3)	25 (62.5)	0.418
History of family CAD, n (%)			27 (14.9)	23 (16.3)	4 (10.0)	0.323
Hypertension, n (%)			124 (68.5)	94 (66.7)	30 (75.0)	0.317
Hyperlipidemia, n (%)			39 (21.5)	34 (24.1)	5 (12.5)	0.115
Diabetes mellitus, n (%)			53 (29.3)	36 (25.5)	17 (42.5)	0.037
Previous MI, n (%)			27 (14.9)	20 (14.2)	7 (17.5)	0.603
Previous coronary intervention, n (%)			19 (10.5)	11 (7.8)	8 (20.0)	0.054
The Killip classification, n (%)						0.004
	I–II		164 (90.6)	133 (94.3)	31 (77.5)	
	III–IV		17 (9.4)	8 (5.7)	9 (22.5)	
GRACE score			125.08 ± 38.51	121.65 ± 37.62	137.20 ± 39.61	0.024
Biochemical parameters						
	NT-proBNP, ng/L		1050.00 [409.00, 3280.00]	838.00 [396.00, 2280.00]	2650.00 [851.00, 6197.50]	<0.001
	eGFR, mL/min/1.73 m^2^		83.52 ± 33.04	90.85 ± 31.60	71.05 ± 31.89	0.001
	TC, mmol/L		4.36 [3.48, 5.14]	4.31 [3.61, 4.99]	4.52 [3.37, 5.27]	0.928
	LDL-C, mmol/L		2.54 ± 0.98	2.54 ± 0.92	2.54 ± 1.19	0.971
	Troponin I, ng/mL		1.00 [0.34, 2.90]	1.00 [0.35, 2.95]	0.95 [0.20, 1.82]	0.272
Echocardiography						
	LVEDd, mm		47.00 [44.00, 51.00]	47.00 [44.00, 51.00]	50.00 [46.00, 56.00]	0.004
	LVESd, mm		28.99 ± 4.03	29.11 ± 3.65	28.38 ± 5.75	0.559
	LVEF, %		56.00 [47.00, 65.00]	59.00 [51.00, 66.00]	47.00 [38.75, 58.00]	<0.001
	LVGLS, %		–15.00 [–18.30, –11.80]	–16.10 [–18.90, –13.50]	–11.00 [–13.70, –7.77]	<0.001
Coronary arteriography						
	Main epicardial coronary arteries ≥70% stenosis					0.530
		1	27 (15.2)	21 (15.0)	6 (15.8)	0.904
		2	48 (27.0)	43 (30.7)	5 (13.2)	0.031
		3	97 (54.5)	70 (50.0)	27 (71.1)	0.021
	Left main ≥50% stenosis		35 (19.7)	26 (18.6)	9 (23.7)	0.482
Therapeutics						
	Revascularization, n (%)					0.627
		PCI	150 (82.9)	116 (82.3)	34 (85.0)	0.686
		CABG	4 (2.2)	3 (2.1)	1 (2.5)	1.000
	Aspirin, n (%)		179 (98.9)	140 (99.3)	39 (97.5)	0.394
	P2Y12 inhibitor (clopidogrel or ticagrelor), n (%)		180 (99.4)	140 (99.3)	40 (100.0)	1.000
	ACEI/ARB/ARNI, n (%)		164 (90.6)	131 (92.9)	33 (82.5)	0.092
	β-blocker, n (%)		169 (93.4)	132 (93.6)	37 (92.5)	1.000
	Statin, n (%)		180 (99.4)	140 (99.3)	40 (100.0)	1.000

Values are expressed as the mean ± standard deviation (SD), median [interquartile range], or n (%). PCI, percutaneous coronary intervention.

### 3.2 Potential Risks in Patients With Events

The analysis revealed no significant differences in sex or body mass index between patients who experienced events and those who did not (*p* > 0.05). However, patients with events had higher levels of N-terminal pro-B-type natriuretic peptide (NT-proBNP) and a greater prevalence of diabetes and multi-vessel coronary disease (MVD) compared with those without events (*p* < 0.05). Additionally, patients with events had worse Killip classification, higher GRACE scores, and reduced eGFR (*p* < 0.05). Furthermore, compared with patients without events, those with events had significantly higher LVEDd and LVGLS, but lower LVEF (*p* < 0.05; Table 1).

To identify new risk factors for predicting the prognosis of patients with NSTEMI, we examined the correlations involving LVGLS. Our analysis revealed that LVGLS positively correlated with log-transformed NT-proBNP (r = 0.535; *p* < 0.001), LVEDd (r = 0.274; *p* < 0.001), and GRACE scores (r = 0.246; *p* < 0.001). Conversely, LVGLS was negatively correlated with LVEF (r = –0.600; *p* < 0.001) and eGFR (r = –0.308; *p* < 0.001; Fig. 2). Moreover, we observed that LVGLS level increased with higher Killip classification (r = 0.394; *p* < 0.001).

**Fig. 2.**
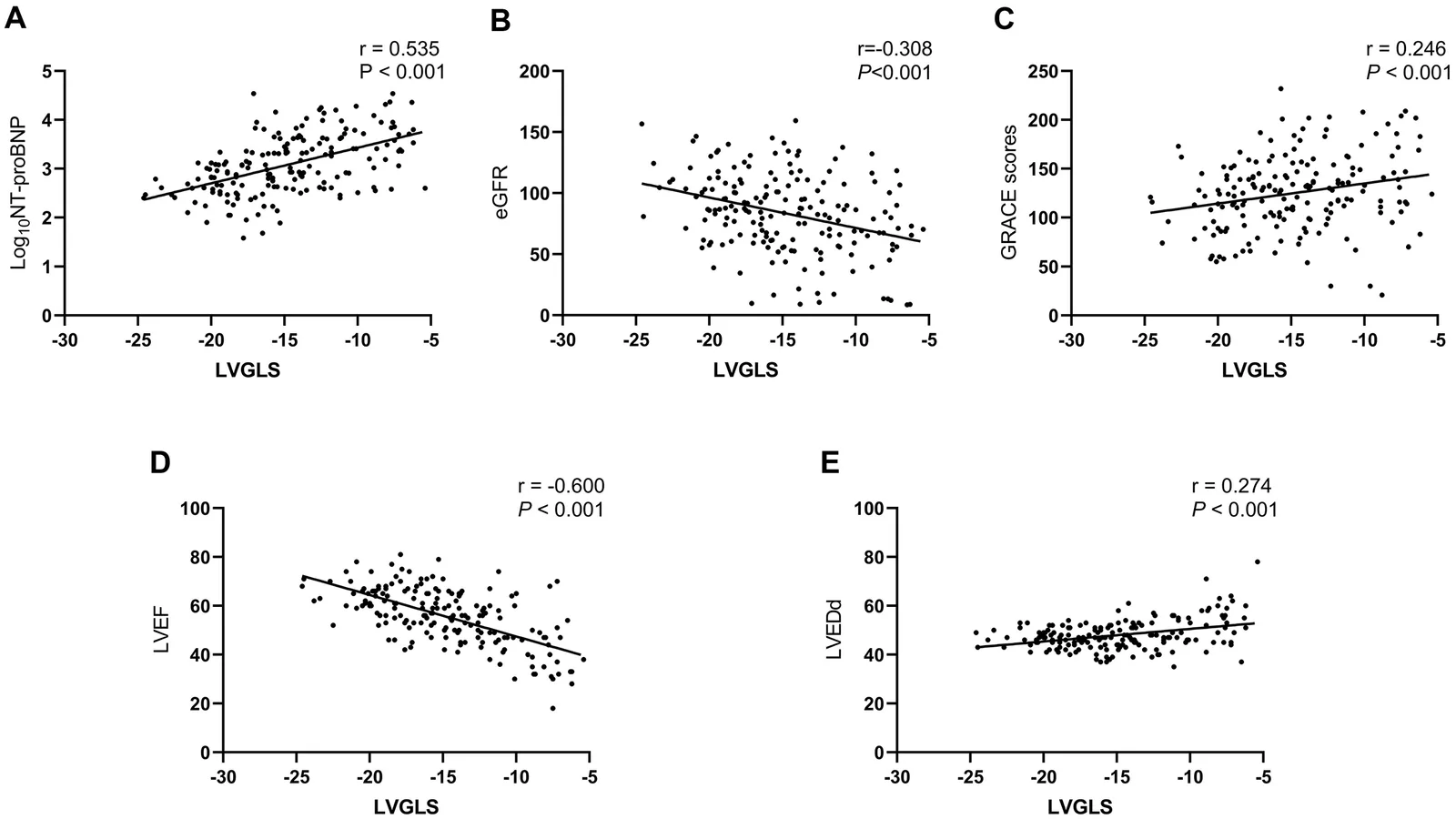
**Correlation analysis**. (A) Correlation between LVGLS and Log10 NT-proBNP. (B) Correlation between LVGLS and eGFR. (C) Correlation analysis between LVGLS and GRACE score. (D) Correlation between LVGLS and LVEF. (E) Correlation between LVGLS and LVEDd.

### 3.3 LVGLS and Clinical Outcomes

Univariate Cox regression analysis identified MVD, diabetes, GRACE score, Killip classification, LVEDd, LVEF, log NT-proBNP, eGFR, and LVGLS as significant prognostic factors in patients with NSTEMI. Subsequently, multivariate analysis was conducted on these factors (*p* < 0.05). These variables revealed that LVGLS level (hazard ratio (HR) = 1.310, 95% confidence interval (CI): 1.172–1.465; *p* < 0.001) independently predicted MACEs in NSTEMI patients (Table 2).

**Table 2.** **Univariate and multivariable Cox analysis**.

	Univariable analysis			Multivariable analysis		
	HR	95% CI	*p*-value	HR	95% CI	*p*-value
Main epicardial coronary arteries ≥70% stenosis = 3	2.318	1.149–4.676	0.019			
Diabetes mellitus	1.974	(1.049–3.679)	0.035			
GRACE score	1.010	(1.001–1.018)	0.023			
Killip classification	2.933	(1.395–6.165)	0.005			
LVEDd	1.077	(1.034–1.121)	<0.001			
LVEF	0.945	(0.923–0.967)	<0.001			
Log_10_ NT-proBNP	2.787	(1.636–4.745)	<0.001			
eGFR	0.983	(0.973–0.993)	<0.001			
LVGLS	1.316	(1.209–1.433)	<0.001	1.310	(1.172–1.465)	<0.001

Since LVGLS and LVEF were strongly correlated, multicollinearity was further evaluated in sensitivity analyses. The VIF values were 20.52 for both LVGLS and LVEF, indicating substantial collinearity. The baseline plus LVEF model yielded a c-index of 0.73, whereas the baseline plus LVGLS model showed a c-index of 0.81. When both LVGLS and LVEF were entered simultaneously, LVGLS remained independently associated with MACE (HR 1.30, 95% CI 1.18–1.45; *p* < 0.001), whereas LVEF was no longer significant (HR 1.00, 95% CI 0.97–1.03; *p* = 0.92) (**Supplementary Table 1**).

ROC analysis was conducted to evaluate the predictive value of LVGLS for MACEs in these patients. The analysis showed that the area under the ROC (AUROC) curves for NT-proBNP, LVEDd, and LVGLS was 0.681 (*p* < 0.001, 95% CI: 0.585–0.776), 0.649 (*p* < 0.05, 95% CI: 0.549–0.750), and 0.809 (*p* < 0.001, 95% CI: 0.731–0.887), respectively. DeLong’s test further compared these AUROC values, revealing that the AUROC for LVGLS was significantly higher than those for NT-proBNP and LVEDd in predicting MACEs in NSTEMI patients (*p* < 0.01). The sensitivity and specificity of LVGLS were 77.5% and 74.5%, respectively (Table 3; Fig. 3).

**Table 3.** **The receiver operating characteristic analysis of important indicators**.

Variables	AUC (95% CI)	Sensitivity/specificity	Cut-off value	*p*-value
NT-proBNP	0.681 (0.585–0.776)	60.00%/75.18%	2285	<0.001
LVEDd	0.649 (0.549–0.750)	40.00%/85.82%	52.5	0.004
LVGLS	0.809 (0.731–0.887)	77.50%/74.47%	–13.75	<0.001

**Fig. 3.**
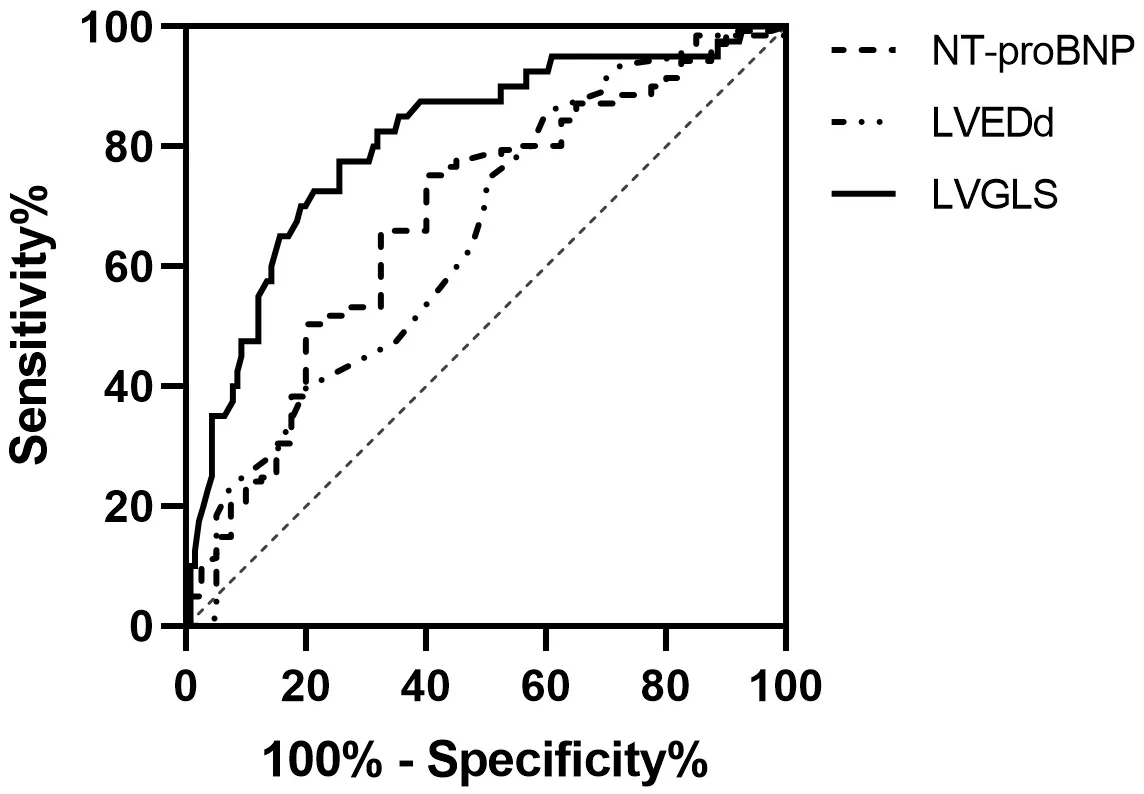
**Receiver operating characteristic curve of the sensitivity and specificity of LVGLS, LVEDd, and NT-proBNP**.

Adding LVGLS to the baseline clinical model improved prognostic performance. The c-index increased from 0.67 to 0.81. For 12-month risk prediction, the AUC increased from 0.668 to 0.835 (delta AUC 0.17; bootstrap 95% CI 0.07–0.28; *p* < 0.001). The continuous NRI was 0.96, and the IDI was 0.22; both remained significant in bootstrap analyses (continuous NRI bootstrap mean 0.99, 95% CI 0.59–1.38; *p* < 0.001; IDI bootstrap mean 0.25, 95% CI 0.12–0.39; *p* < 0.001) (**Supplementary Tables 2**,**3**; **Supplementary Fig. 1**).

The original Youden index-derived optimal LVGLS cutoff was –13.70%, which was very close to the prespecified threshold of –13.75%. Bootstrap validation showed a mean cutoff of –13.26%, a median of –13.70%, and a 95% CI of –14.90% to –11.50%. The bootstrap mean AUC was 0.810 (95% CI 0.73–0.88), indicating acceptable internal stability of prognostic performance (**Supplementary Table 3**; **Supplementary Fig. 2**).

The Kaplan–Meier curve demonstrated that patients with LVGLS greater than –13.75% had a significantly higher risk of MACEs than those with LVGLS ≤–13.75% (HR 7.463, 95% CI 3.831–14.540; *p* < 0.001; Fig. 4).

**Fig. 4.**
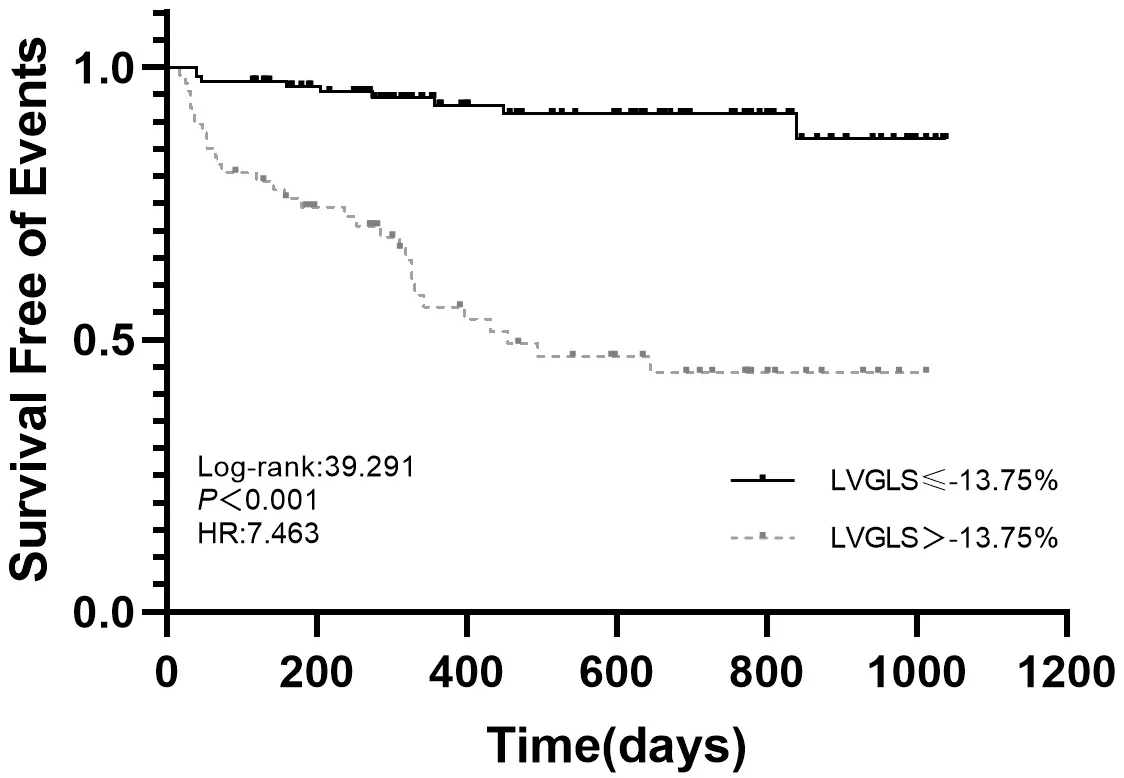
**Kaplan–Meier curve for event-free survival stratified by LVGLS**.

When LVGLS was analyzed as a continuous variable, the association with MACEs remained significant in the Cox regression model. Exploratory restricted cubic spline analysis did not provide clear evidence of marked nonlinearity, supporting the use of LVGLS as a continuous predictor in this cohort (**Supplementary Fig. 3**).

No significant interaction was observed between LVGLS and diabetes (HR 1.03, 95% CI 0.86–1.25; *p* = 0.73). In sex-stratified analyses, LVGLS remained significantly associated with MACE in men (HR 1.33, 95% CI 1.20–1.47; *p* < 0.001), whereas the association in women did not reach statistical significance (HR 1.11, 95% CI 0.90–1.38; *p* = 0.33). The LVGLS × sex interaction was not statistically significant (HR 0.87, 95% CI 0.71–1.07; *p* = 0.18) (**Supplementary Fig. 4**; **Supplementary Table 1**).

## 4. Discussion

This study evaluated the prognostic significance of LVGLS in 181 patients with NSTEMI. Our findings revealed that LVGLS worsened with increasing Killip class, correlated negatively with LVEF, and correlated positively with NT-proBNP. Further analysis showed that LVGLS could independently predict adverse outcomes in patients with NSTEMI after adjustment for clinical risk factors, laboratory findings, and echocardiographic parameters. Notably, patients with LVGLS >–13.75% had a 7.463-fold higher risk of MACEs than those with LVGLS ≤–13.75%.

The dominant prognostic contribution of LVGLS compared with LVEF may reflect the greater sensitivity of longitudinal strain to early myocardial injury. In the presence of substantial collinearity, LVGLS retained prognostic significance. In contrast, LVEF did not, suggesting that LVGLS captured the more clinically relevant component of systolic dysfunction for risk stratification in this cohort [7,8]. These findings indicate that LVGLS provides incremental prognostic information beyond conventional clinical predictors, as reflected by improved discrimination and reclassification.

The prognosis of patients with NSTEMI is influenced by multiple interacting factors. Previous studies have shown that initial cardiac troponin levels, eGFR, natriuretic peptide levels, and GRACE scores each provide prognostic information [3]. In addition to circulating biomarkers, several studies have evaluated the role of two-dimensional echocardiography in post-NSTEMI risk stratification. Among non-invasive assessments, LV systolic function is a key prognostic marker. Currently, LVEF is the most widely used measure of LV systolic function. However, LVEF is commonly derived using the Simpson method, which requires high-quality images, is time-consuming, and may have limited reproducibility [4,5]. To overcome these limitations and improve prognostic assessment, LVGLS has been introduced as a quantitative measure of LV systolic function. Since LVGLS is less operator-dependent, it generally shows lower interobserver variability than LVEF [16]. The accuracy of LVGLS has been validated against sonomicrometry and tagged magnetic resonance imaging [17]. LVGLS uses two-dimensional speckle-tracking imaging to follow myocardial acoustic markers throughout the cardiac cycle and to semi-automatically quantify myocardial deformation with the automated function imaging (AFI) tool. Since this technique is less dependent on the insonation angle relative to myocardial motion, it improves the reproducibility of quantitative assessment of LV systolic function. In addition, the technique is practical at the bedside and relatively inexpensive.

LVGLS has become a crucial metric in cardiovascular diagnostics, risk stratification, and prognostic evaluation. It enables precise quantification of left ventricular systolic function and has demonstrated significant diagnostic and prognostic utility across various cardiovascular conditions, including heart failure, cardiomyopathies, valvular heart disease, and cardio-oncology [18,19,20,21,22]. Unlike traditional echocardiographic measures such as LVEF and WMSI, LVGLS can detect early left ventricular systolic dysfunction that conventional metrics may miss.

Although data on the prognostic role of LVGLS in NSTEMI patients are limited, existing research strongly supports its potential as a prognostic marker. A prospective study by Ersbøll et al. [23] involving 988 AMI patients, 31.5% of whom had NSTEMI, identified LVGLS as an independent predictor of sudden cardiac death and ventricular arrhythmias post-AMI. Another study of 849 AMI patients, 32% with NSTEMI and LVEF >40%, reported that patients with LVGLS >–14% had a threefold higher risk of endpoint events, including all-cause death and heart failure rehospitalization, compared with those with LVGLS ≤–14%, consistent with the cutoff values observed in the present study [24]. A previous study demonstrated a negative correlation between LVGLS and LVEF in 44 patients with NSTEMI and 20 healthy controls, aligning with our findings [25]. Another study identified baseline LVGLS ≥–12% and a lack of LVGLS improvement post-percutaneous coronary intervention (PCI) as independent predictors of left ventricular remodeling at 6 months in 70 patients with NSTEMI [26]. In our study, LVGLS negatively correlated with LVEF and positively correlated with LVEDd, consistent with more advanced ventricular dysfunction and adverse left ventricular remodeling, and this finding was in line with previous research. Haugaa et al. [27] conducted a prospective study of 301 NSTEMI patients and found that LVGLS, but not LVEF, predicted ventricular arrhythmia.

Our study showed that baseline LVGLS measured after NSTEMI significantly predicted MACEs and remained independently associated with outcomes after adjustment for a broad range of clinical and echocardiographic variables. In multivariable Cox regression, LVGLS demonstrated stronger prognostic relevance than other biomarkers and echocardiographic parameters. These findings support the clinical value of LVGLS for risk stratification in NSTEMI.

## 5. Study Limitations

This study has several limitations. First, as a single-center study with a relatively small sample size and a limited number of MACEs, there is a risk of selection bias, which may compromise the stability of multivariable analyses. Bootstrap resampling showed that the cohort-derived LVGLS threshold was reasonably stable; however, the confidence interval around the cutoff remained relatively wide, which is consistent with the modest sample size of this cohort. Second, although the optimal threshold analysis was retained for clinical interpretability and Kaplan–Meier visualization, deriving and testing the cutoff in the same dataset may introduce optimism bias. To address this concern, we additionally analyzed LVGLS as a continuous variable and performed an exploratory spline-based sensitivity analysis. Third, post hoc power was adequate for the primary LVGLS–MACE association, but statistical power remained limited for rare endpoint components and subgroup analyses. Fourth, only baseline LVGLS at admission was measured, with no assessments conducted post-PCI treatment or at discharge. Consequently, larger prospective multicenter studies with serial LVGLS measurements are necessary to validate these findings.

## Data Availability

The datasets generated or analyzed during the current study are available from the corresponding author and the first author upon reasonable request. Due to ethical restrictions related to patient confidentiality and the policies of the institutional review board (IRB) of our hospital, raw clinical and echocardiographic data cannot be publicly shared. However, de-identified data can be made available from the corresponding author upon reasonable request, provided that the request is accompanied by a research proposal approved by the applicant’s institutional ethics committee. Additionally, key summary statistics and figures supporting the main findings of this study are included in the manuscript for transparency and reproducibility.

## 6. Conclusions

LVGLS is a strong predictor of MACEs in patients with NSTEMI and may facilitate early risk stratification.

## Abbreviations

LVGLS, Left ventricular global longitudinal strain; NSTEMI, non-ST-segment elevation myocardial infarction; MACE, Major adverse cardiovascular events; BMI, body mass index; CAD, coronary artery disease; MI, myocardial infarction; GRACE, Global Registry of Acute Coronary Events; NT-proBNP, N-terminal pro-B type natriuretic peptide; eGFR, estimated glomerular filtration rate; TC, total cholesterol; LDL-C, low-density lipoprotein cholesterol; LVEDd, left ventricular end diastolic diameter; LVESd, left ventricular end systolic diameter; LVEF, left ventricular ejection fraction; PCI, percutaneous coronary intervention; CABG, coronary artery bypass grafting; ACEI, angiotensin-converting enzyme inhibitor; ARB, angiotensin II receptor blocker; ARNI, angiotensin receptor-neprilysin inhibitor.
