# Supplementary material for: Outcome Stratification in Non-ST-Segment Elevation Myocardial Infarction: Standardized Assessment of Left Ventricular Global Longitudinal Strain
Source: Rev Cardiovasc Med. 2026 Sep 16;27(9):49528. doi: 10.31083/RCM49528 (PMC13653160; doi:10.31083/RCM49528)
Supplement: Supplementary file 1 [file 2153-8174-27-9-49528-s1.zip › Supplementary Material.docx]

***RCM 49528***

***Title:* Outcome Stratification In Non-ST-Segment Elevation Myocardial Infarction: Standardized Assessment Of Left Ventricular Global Longitudinal Strain**

**Supplementary Materials**

This supplementary file provides additional figures and tables supporting the incremental prognostic value, internal validation, interaction analyses, and power context.

**Supplementary Fig. 1. ROC curves for 12-month MACE prediction**. Receiver operating characteristic curves comparing the baseline clinical model (GRACE score, diabetes, and Killip classification) with the extended model including LVGLS.

**Supplementary Fig. 2. Bootstrap distribution of the LVGLS cutoff**. Histogram of bootstrap-derived LVGLS cutoffs showing the stability of the Youden-derived threshold. The solid red line indicates the observed cutoff, and the dashed green line indicates the reported cutoff of -13.75%.

**Supplementary Fig. 3. Restricted cubic spline of LVGLS and MACE risk**. Restricted cubic spline analysis illustrating the dose-response relationship between LVGLS and MACE risk. No clear evidence of marked nonlinearity was observed in this cohort.

**Supplementary Fig. 4. Forest plot of LVGLS effect estimates**. Hazard ratio estimates for LVGLS in the overall model, sex-stratified models, and interaction analyses.

# Supplementary Table 1. Cox regression models.

| Model | covariate | coef | HR | SE | p | HR_95L | HR_95U |
| --- | --- | --- | --- | --- | --- | --- | --- |
| Main model | grace | 0.0 | 1.0 | 0.0 | 0.67 | 0.99 | 1.01 |
| Main model | diabetes | 0.14 | 1.15 | 0.32 | 0.66 | 0.61 | 2.17 |
| Main model | killip_high | 0.24 | 1.27 | 0.38 | 0.52 | 0.61 | 2.67 |
| Main model | gls | 0.26 | 1.3 | 0.05 | 0.0 | 1.19 | 1.42 |
| Diabetes interaction model | grace | 0.0 | 1.0 | 0.0 | 0.66 | 0.99 | 1.01 |
| Diabetes interaction model | diabetes | 0.5 | 1.65 | 1.09 | 0.64 | 0.2 | 13.9 |
| Diabetes interaction model | killip_high | 0.25 | 1.29 | 0.38 | 0.5 | 0.61 | 2.71 |
| Diabetes interaction model | gls | 0.25 | 1.29 | 0.05 | 0.0 | 1.16 | 1.43 |
| Diabetes interaction model | gls_x_diabetes | 0.03 | 1.03 | 0.1 | 0.73 | 0.86 | 1.25 |
| Sex interaction model | grace | 0.0 | 1.0 | 0.0 | 0.85 | 0.99 | 1.01 |
| Sex interaction model | diabetes | 0.21 | 1.23 | 0.33 | 0.53 | 0.64 | 2.34 |
| Sex interaction model | killip_high | 0.35 | 1.42 | 0.38 | 0.35 | 0.68 | 2.99 |
| Sex interaction model | gls | 0.29 | 1.33 | 0.05 | 0.0 | 1.2 | 1.48 |
| Sex interaction model | female | -1.93 | 0.14 | 1.33 | 0.15 | 0.01 | 1.97 |
| Sex interaction model | gls_x_female | -0.14 | 0.87 | 0.1 | 0.18 | 0.71 | 1.07 |
| Male subgroup | grace | 0.0 | 1.0 | 0.01 | 0.59 | 0.99 | 1.01 |
| Male subgroup | diabetes | 0.24 | 1.27 | 0.38 | 0.53 | 0.6 | 2.68 |
| Male subgroup | killip_high | 0.25 | 1.29 | 0.41 | 0.54 | 0.58 | 2.87 |
| Male subgroup | gls | 0.28 | 1.33 | 0.05 | 0.0 | 1.2 | 1.47 |
| Female subgroup | grace | -0.01 | 0.99 | 0.01 | 0.52 | 0.97 | 1.02 |
| Female subgroup | diabetes | 0.29 | 1.34 | 0.77 | 0.71 | 0.29 | 6.09 |
| Female subgroup | killip_high | 1.06 | 2.89 | 1.11 | 0.34 | 0.33 | 25.55 |
| Female subgroup | gls | 0.11 | 1.11 | 0.11 | 0.33 | 0.9 | 1.38 |

Detailed coefficients, hazard ratios, confidence intervals, and p-values for the main, interaction, and subgroup Cox models.

# Supplementary Table 2. Incremental prognostic value of LVGLS.

| Model | N | Events | C_index | AUC_12m | Delta_AUC_12m | Continuous_NRI | IDI |
| --- | --- | --- | --- | --- | --- | --- | --- |
| Base model | 181 | 40 | 0.67 | 0.67 | nan | nan | nan |
| Base + LVGLS | 181 | 40 | 0.81 | 0.84 | 0.17 | 0.96 | 0.22 |

Summary of model discrimination before and after adding LVGLS to the baseline clinical model.

# Supplementary Table 3. Bootstrap validation and reclassification metrics.

| Metric | Observed | Bootstrap_mean | CI_95_L | CI_95_U | P_bootstrap |
| --- | --- | --- | --- | --- | --- |
| Delta AUC | 0.17 | 0.171 | 0.07 | 0.28 | 0.0 |
| Continuous NRI | 0.96 | 0.99 | 0.59 | 1.38 | 0.0 |
| IDI | 0.22 | 0.25 | 0.12 | 0.39 | 0.0 |
| LVGLS cutoff | -13.7 | -13.26 | -14.9 | -11.5 | nan |

Bootstrap-derived estimates for delta AUC, continuous NRI, IDI, and LVGLS cutoff stability.

# Supplementary Table 4. Post-hoc power context.

| Endpoint | Events | LVGLS_threshold_HR | Threshold_positive_proportion | Approx_post_hoc_power |
| --- | --- | --- | --- | --- |
| Composite MACE | 40 | 6.46 | 0.37 | 1.0 |

Event-based post-hoc power context for the primary LVGLS threshold analysis.
